# Role of Mean Platelet Volume in the Prognosis of Gallbladder Carcinoma: A Tertiary Centre Experience

**DOI:** 10.7759/cureus.16389

**Published:** 2021-07-14

**Authors:** Prakash BV, Ali Z Anwar, Raghavendra Harsha, Praveen r Arakeri, Pavan Jonnada

**Affiliations:** 1 Surgical Oncology, Kidwai Memorial Institute of Oncology, Bengaluru, IND

**Keywords:** gall bladder cancer, diagnosis, lymph node status, overall stage, mean platelet volume

## Abstract

Mean platelet volume (MPV) is an inflammatory marker indicative of platelet activation. There are several studies that suggest an association between the neoplastic process and cancer metastasis. We performed a retrospective analysis to investigate the role of MPV as a prognostic informative marker in gallbladder cancer. This study included 73 patients who underwent treatment for gallbladder cancer with curative or palliative intent. MPV was obtained and statistically analysed to investigate the association between the nodal status (N), the overall stage as per the American Joint Committee on Cancer (AJCC) staging system, perineural invasion, and differentiation of the tumor. The statistical analysis was done using SPSS Statistics, version 23 (IBM Corp., Armonk, NY). We found that the MPV values were significantly high in node-positive cases (OR = 3.623, 95% CI = 7.778-1.687, p value = −0.0001), cases in the advanced stage (OR = 3.623, 95% CI = 7.778-1.687, p value = 0.0001), cases with perineural invasion (OR = 3.396, 95% CI = 8.319-1.387, p value = −0.0001), and poor differentiation (OR = 2.327, 95% CI = 4.651-1.164, p value = −0.002 ). MPV is an inexpensive and convenient inflammatory marker that correlates with nodal positivity in the staging and prognostication of gallbladder cancer. This marker can be used to ascertain the risk status of gallbladder cancer.

## Introduction

Gallbladder cancer is the most common cancer of the hepatobiliary system, with an incidence of 1.2% of the total cancer diagnoses, accounting for approximately 165,087 deaths and 1.7% of the total cancer deaths in 2018 [1].

The presentation of gallbladder cancer is often confusing, which causes delay in diagnosis. It is often discovered incidentally after a simple cholecystectomy or when it causes ascites or jaundice at a very advanced stage [2]. It tends to be unresectable, with a dismal prognosis at stages I, II, III, and IV (60%, 50%, 20%-25%, and 5%-15%, respectively) [3].

Surgery can provide a complete cure when performed in the early stage of the disease, with simple cholecystectomy sufficing for in situ carcinoma or T1a, with a more radical resection needed in the advanced stage if possible where a negative margin is to be obtained, requiring a resection of the liver and bile ducts via a local lymphadenectomy [3,4].

There is limited availability of tumor markers that can be employed in the diagnosis of gallbladder cancer, with carcinoma embryonic antigen (CEA) and carbohydrate antigen (CA) 19-9 being the two most commonly used markers [5]. Other markers, which are not generally used, are CA 15-3, CA 242, and Mac-2BP. However, these have been found to have variable sensitivity and specificity [5,6].

## Materials and methods

For this study, we retrospectively analysed data on patients with gallbladder cancer at the Kidwai Memorial Institute of Oncology, Bengaluru, India, between January 2018 and January 2021. Data on 73 patients, from the computer database of the institute, was utilized, including data on the staging of the patients according to American Joint Committee on Cancer (AJCC) recommendations and the histological characteristics of the tumor afflicting the patients. The institutional review board clearance was obtained with the proper consent.

For the analysis of mean platelet volume (MPV), a hemogram was obtained from the blood collected, approximately 5 to 10 ml from a peripheral vein into sterilized ethylenediaminetetraacetic acid (EDTA) tubes, from the patients. The blood reports were collected in the morning to minimize circadian rhythm effects, and the MPV value considered was the value at the time of diagnosis of the patient.

The statistical analysis was performed using the SPSS Statistics, version 23 (IBM Corp., Armonk, NY). The parameters were compared using means and standard deviations, and the parametric variables were compared using chi-square analysis. A receiver-operating characteristic (ROC) curve analysis was performed to identify the optimal cutoff values for MPV. A p value of <0.05 was considered significant.

## Results

This study included 73 patients with gallbladder cancer, comprising 49 females and 24 males in the age range of 38 to 82 years, with a mean age of 60.2 years. The patient characteristics are presented in Table 1.

**Table 1:** Characteristics of study participants AJCC, American Joint Committee on Cancer

Baseline characters	
Number of patients	73
Males	24
Females	49
Age range	38-82 years
Mean age	60.2 years
Mean platelet volume range	7.30-11.83 fl
Mean platelet volume (mean)	9.88 fl
AJCC stage I	9
AJCC stage II	11
AJCC stage III	29
AJCC stage IV	24
Poor differentiation	43
Well, moderate differentiation	30
With perineural invasion	40
Without perineural invasion	33

**Figure 1:**
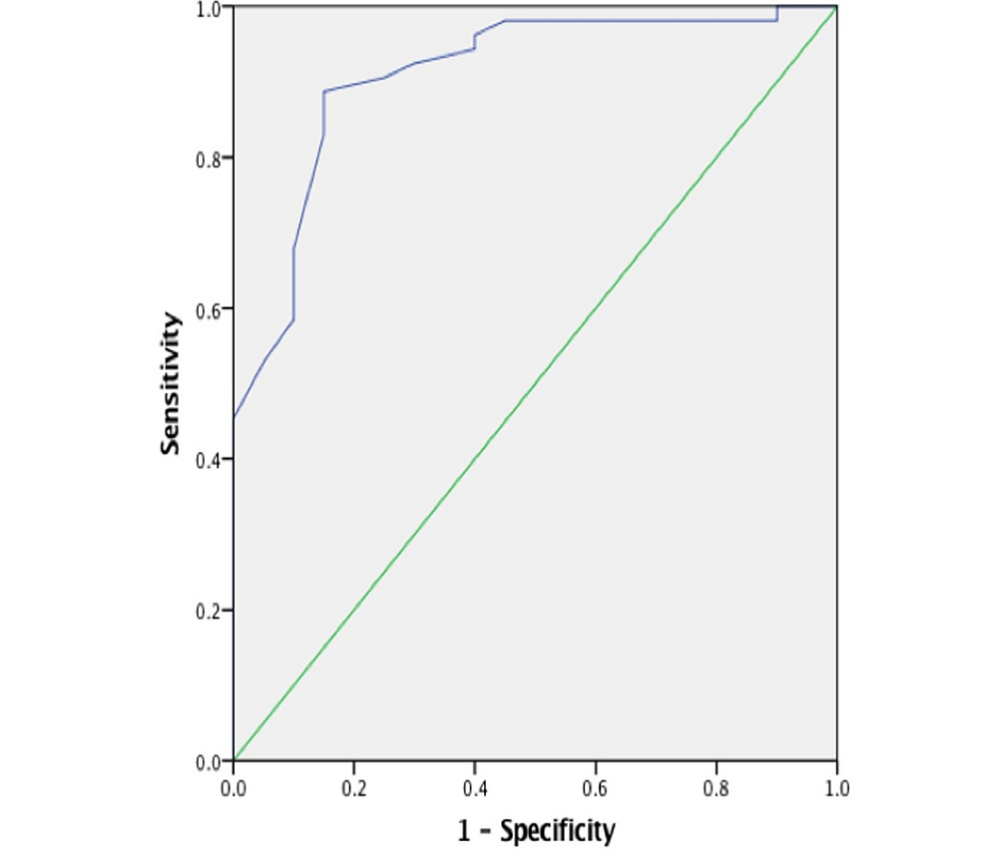
Receiver-operating characteristic curve analysis showing the mean platelet volume cutoff

The area under curve was 0.909 for MPV (Figure 1).

The patients were then divided into two groups: one with a low MPV (<9.4), and the other with a high MPV (≥9.4).

**Figure 2:**
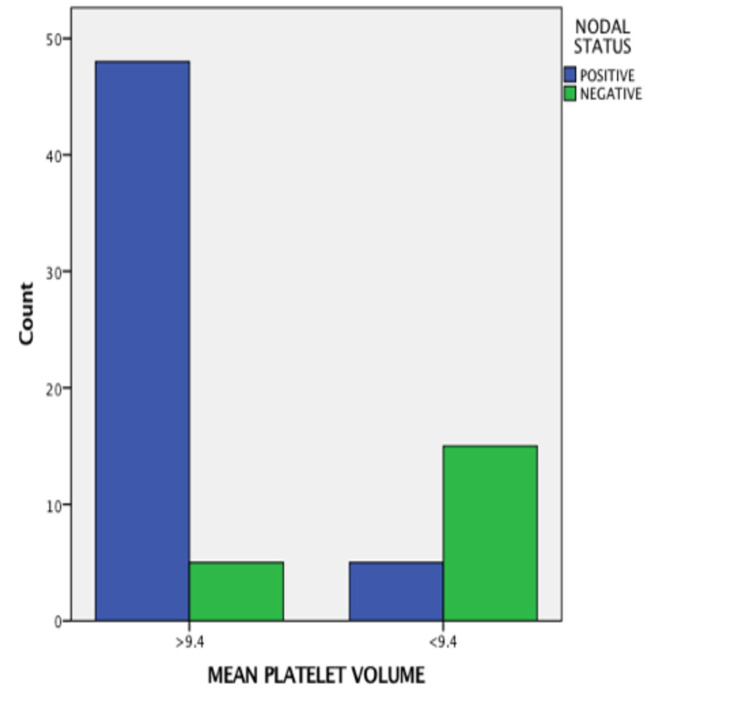
Relationship between mean platelet volume and nodal status

**Figure 3:**
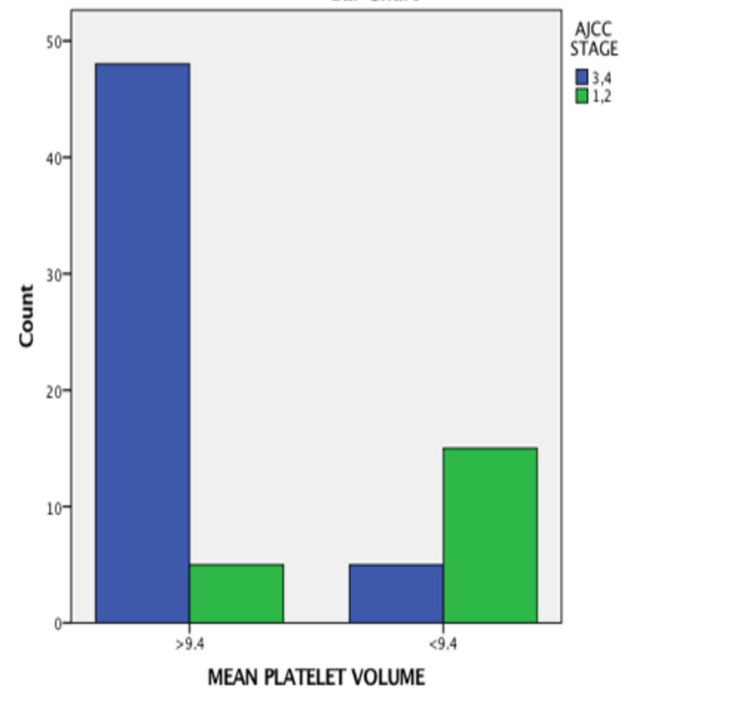
Relationship between mean platelet volume and American Joint Committee on Cancer stage

**Figure 4:**
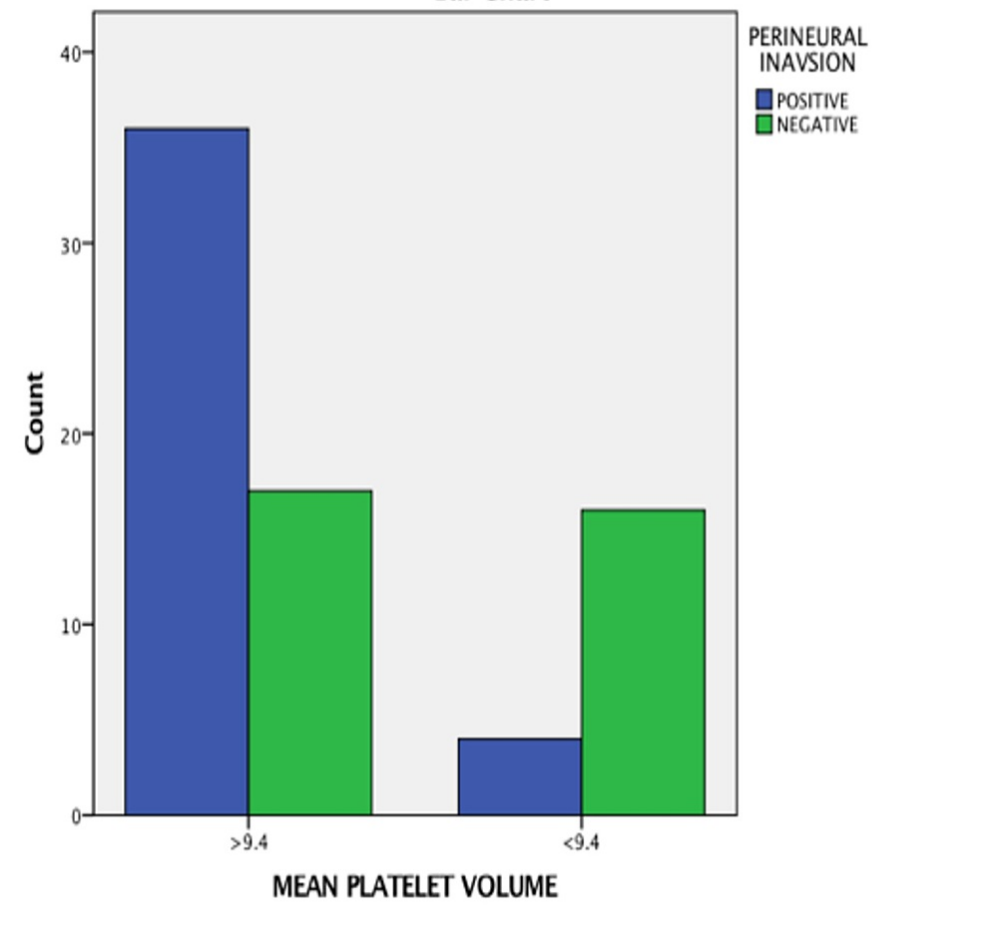
Relationship between mean platelet volume and perineural invasion

**Figure 5:**
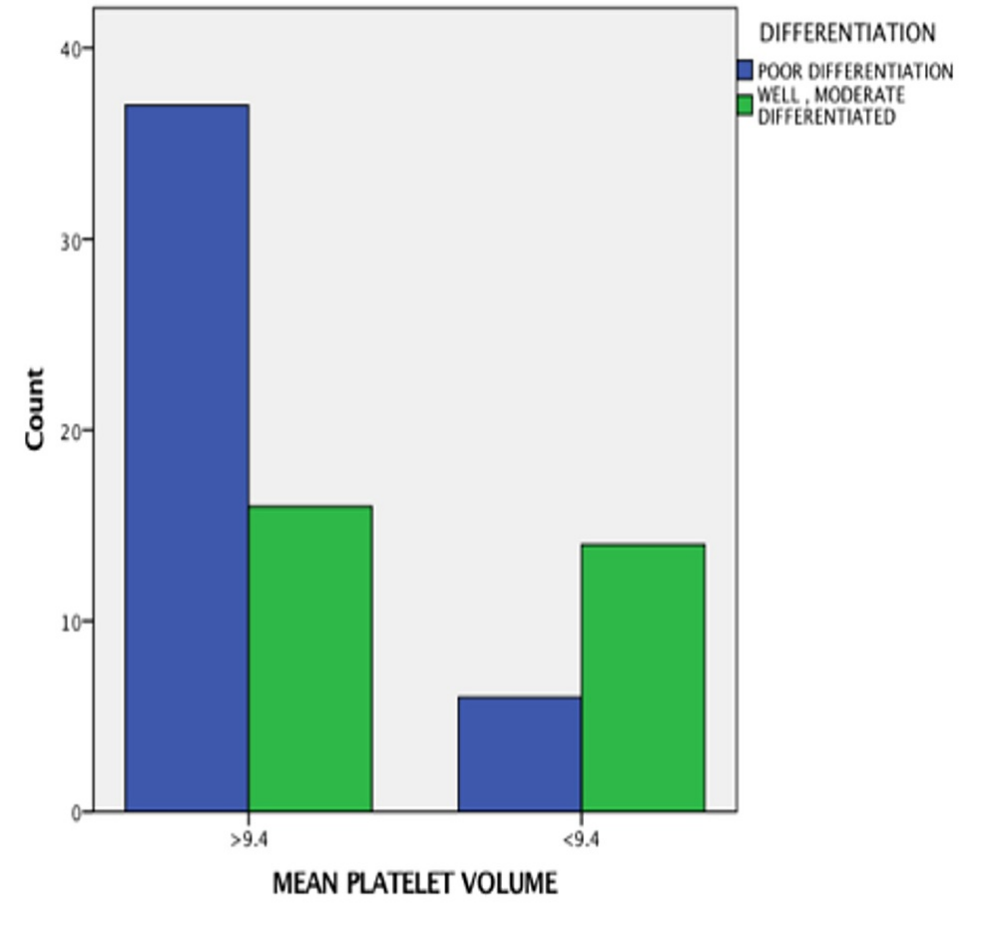
Relationship between mean platelet volume and differentiation

The MPV values were significantly high in node-positive cases (OR = 3.623, 95% CI = 7.778-1.687, p value = −0.0001), as shown in Figure 2; advanced stage cases (OR = 3.623, 95% CI = 7.778-1.687, p value = 0.0001), as shown in Figure 3; cases with perineural invasion (OR = 3.396, 95% CI = 8.319-1.387, p value = −0.0001), as shown in Figure 4; and cases with poor differentiation (OR = 2.327, 95% CI = 4,651-1.164, p value = −0.002 ), as shown in Figure 5. There was no significant correlation between high MPV and the age or sex of the patient. There was a negative correlation between high MPV and the age or sex of the patient, as shown in Table 2.

**Table 2:** Relationship between MPV and demographic and clinical parameters AJCC, American Joint Committee on Cancer; MPV, mean platelet volume

	No. of patients	Low MPV (<9.4 fl)	High MPV (≥9.4 fl)	Chi-square	p value	Odds ratio (95% CI)
Gender				2.07	0.15	0.77 (1.054-0.575)
Male	24	5	19			
Female	49	14	35			
Age				1.77	0.18	1.30 (2.34-0.81)
<60 years	31	11	20			
>60 years	42	9	33			
AJCC stage				31.38	0.0001	3.623 (7.778-1.687)
Stage I, II	20	15	5			
Stage III, IV	53	5	48			
Nodal status				31.38	0.0001	3.623 (7.778-1.687)
Node negative	20	15	5			
Node positive	53	5	48			
Perineural invasion				13.464	0.0001	3.396 (8.319-1.387)
Perineural invasion present	40	4	36			
Perinueral invasion absent	33	16	17			
Differentiation				9.507	0.002	2.327 (4.651-1.164)
Poor differentiation	43	6	37			
Well, moderate differentiation	30	14	16			

## Discussion

In this study, it was observed that an increased MPV value can reliably predict the involvement of lymph nodes in cases of gallbladder cancer. This research shows that MPV is a promising marker that aids in the prediction of advanced stage, perineural invasion, as well as poor differentiation histological characteristics in cases of gallbladder cancer.

An increase in MPV values is generally regarded as the clumping of platelets, which is one of the features of the inflammation processes, which enables the oncogenesis via generation of genetic-material-damaging agents like reactive oxygen species and promotes dissemination and invasion of cancer cells via production of chemokines and various other agents. The increased MPV can also lead to increased platelet depletion and indicates that immature platelets are being released into circulation, which are larger in size than normal platelets [7-10].

Several studies have found a correlation between high MPV values in different cancers. An MPV value higher than 8.25 fl in cases of gastric carcinoma is useful for monitoring patients’ risk of gastric carcinoma [11]. In cases of ovarian carcinoma, an MPV value higher than 8.26 fl is correlated with a worse tumor burden and prognosis [12]. In this study, an MPV value greater than 9.4 fl was found to be correlated with worse prognosis, including worse histological features and increased nodal dissemination, as seen in a similar study [13].

However, in a study conducted by Sun et al., it was found that low MPV values of less than 8.10 fl are correlated with a worse prognosis in cases of esophageal carcinoma [13]. However, in other studies including cases of gastric carcinoma, it was found that increased MPV values greater than 10.2 fl were correlated with a worse prognosis and lymph node metastasis [14-16].

This study also had some limitations. It was retrospective in nature and was based on case records; the details pertaining to each case were limited in nature. Furthermore, some patients underwent chemotherapy, which may have influenced the attributes of the disease.

This study proves conclusively that there is a correlation between increased MPV values and the local dissemination and prognosis of gallbladder cancer. Although MPV has low specificity at low values, it is a noninvasive, inexpensive marker that can be an invaluable addition to the present repertoire of tumor markers for risk stratification and predicting the prognosis of gallbladder cancer.

## Conclusions

The evaluation and procuring of MPV is quick and inexpensive, making it useful for staging and risk assessment, in addition to being an inflammatory marker. The MPV value of 9.4 fl is the cutoff for predicting nodal metastasis, advanced stage, and worse histological features such as poor differentiation and perineural invasion. Hence, the inclusion of this parameter can facilitate determining the prognosis of the disease.
